# Soft Tissue Manipulation Alters RANTES/CCL5 and IL-4 Cytokine Levels in a Rat Model of Chronic Low Back Pain

**DOI:** 10.3390/ijms241814392

**Published:** 2023-09-21

**Authors:** Carmela L. Marciano, Taylor A. Hiland, Krista L. Jackson, Sierra Street, Carson Maris, Andrew Ehrsam, Julia M. Hum, Mary Terry Loghmani, Tien-Min G. Chu, Kyung S. Kang, Jonathan W. Lowery

**Affiliations:** 1 Division of Biomedical Science, College of Osteopathic Medicine, Marian University, Indianapolis, IN 46222, USA; cmarciano@marian.edu (C.L.M.); thiland@marian.edu (T.A.H.); sst694@marian.edu (S.S.); aehrsam088@marian.edu (A.E.); jmhum@marian.edu (J.M.H.); 2 Bone & Muscle Research Group, Marian University, Indianapolis, IN 46222, USA; cmaris@butler.edu (C.M.); kkang@marian.edu (K.S.K.); 3 Indiana Biosciences Research Institute, Indianapolis, IN 46222, USA; 4 Department of Physical Therapy, School of Health and Human Sciences, Indiana University, Indianapolis, IN 46222, USA; mloghman@iu.edu; 5 Indiana Center for Musculoskeletal Health, School of Medicine, Indiana University, Indianapolis, IN 46222, USA; tgchu@iu.edu; 6 Department of Biomedical Sciences and Comprehensive Care, School of Dentistry, Indiana University, Indianapolis, IN 46222, USA; 7 Witchger School of Engineering, Marian University, Indianapolis, IN 46222, USA; 8 Division of Academic Affairs, Marian University, Indianapolis, IN 46222, USA; 9 Department of Orthopaedic Surgery, School of Medicine, Indiana University, Indianapolis, IN 46222, USA

**Keywords:** inflammation, massage, soft tissue manipulation, cytokine, low back pain, musculoskeletal

## Abstract

Low back pain (LBP) is a common musculoskeletal complaint that can impede physical function and mobility. Current management often involves pain medication, but there is a need for non-pharmacological and non-invasive interventions. Soft tissue manipulation (STM), such as massage, has been shown to be effective in human subjects, but the molecular mechanisms underlying these findings are not well understood. In this paper, we evaluated potential changes in the soft tissue levels of more than thirty pro- or anti-inflammatory cytokines following instrument-assisted STM (IASTM) in rats with chronic, induced LBP using Complete Freund’s Adjuvant. Our results indicate that IASTM is associated with reduced soft tissue levels of Regulated on Activation, Normal T cell Expressed and Secreted (RANTES)/Chemokine (C-C motif) ligand 5 (CCL5) and increased soft tissue levels of Interleukin (IL)-4, which are pro-inflammatory and anti-inflammatory factors, respectively, by 120 min post-treatment. IASTM was not associated with tissue-level changes in C-X-C Motif Chemokine Ligand (CXCL)-5/Lipopolysaccharide-Induced CXC Chemokine (LIX)–which is the murine homologue of IL-8, CXCL-7, Granulocyte-Macrophage-Colony Simulating Factor (GM-CSF), Intercellular Adhesion Molecule (ICAM)-1, IL1-Receptor Antagonist (IL-1ra), IL-6, Interferon-Inducible Protein (IP)-10/CXCL-10, L-selectin, Tumor Necrosis Factor (TNF)-α, or Vascular Endothelial Growth Factor (VEGF) at either 30 or 120 min post-treatment. Combined, our findings raise the possibility that IASTM may exert tissue-level effects associated with improved clinical outcomes and potentially beneficial changes in pro-/anti-inflammatory cytokines in circulation and at the tissue level.

## 1. Introduction

Low back pain (LBP) is a common complaint among adults, with approximately 39 percent of Americans experiencing LBP in 2019 [1]. Pharmacological intervention, such as acetaminophen and non-steroidal inflammatory drugs (NSAIDS), is the first-line treatment for LBP [2]. Pharmaceuticals as the primary treatment for LBP can be costly and less accessible, especially for the 44.8 percent of individuals that struggle with back pain and live below the federal poverty level [1]. Additionally, opioids are commonly prescribed for low back pain, and more than half of opioid users have reported LBP despite these medications showing limited efficacy for this condition [3].

Prior work in human subjects indicates that soft tissue manipulation (STM), such as massage, may accelerate the return to function, improve mental and emotional wellbeing, and reduce the need for opioid medication usage [4]. However, the heterogeneity of lifestyles and body conditions, potential co-morbidities, and the inherent mind–body aspect of manual therapies complicate our understanding of the molecular mechanisms underlying these findings.

Instrument-assisted STM (IASTM) is a manual therapy modality that stimulates painful areas of soft tissue with a rigid instrument. A recent study indicated that IASTM improves gait patterns in rats with induced LBP [5], suggesting that IASTM may promote pain relief and/or functional recovery from LBP. This same study revealed that IASTM modulated the serum levels of cytokines involved in pain and the inflammatory response, resulting in increased levels of Neuropeptide-Y (NPY) and reduced levels of Regulated on Activation, Normal T cell Expressed and Secreted (RANTES)/Chemokine (C-C motif) ligand 5 (CCL5) within three and fourteen days of treatment, respectively [5].

Despite these findings, the molecular changes associated with STM in the tissue itself remain uncertain. This knowledge gap may prevent future therapeutic potential and widespread adoption of this non-invasive approach to pain management and inflammation. Thus, we sought to extend prior work on IASTM in a rat model of induced LBP to examine the potential changes in tissue levels of more than thirty pro- or anti-inflammatory cytokines following IASTM. Our results indicate that IASTM is associated with reduced soft tissue levels of RANTES/CCL5 and increased soft tissue levels of IL-4, which are pro-inflammatory and anti-inflammatory cytokines, respectively. These findings advance the mechanistic understanding of tissue-level responses to IASTM and provide rationale for future studies involving STM in human subjects with LBP.

## 2. Results

**Figure 1:**
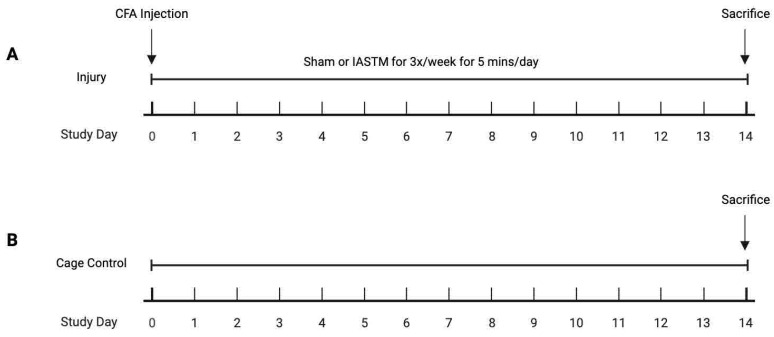
Schematic of study design and timeline. (**A**): For samples in the present study, injury was performed on Day 0 to *n* = 15 rats using an injection of Complete Freund’s Adjuvant (CFA). Rats were then randomly assigned to sham (i.e., untreated) (*n* = 5) or instrument-assisted soft tissue manipulation (IASTM) treatment (*n* = 10) for intervention 3 times per week over two weeks for five minutes per session. On Day 14, IASTM treatment group was further divided into sacrifice within 30 min (*n* = 5) or 2 h (*n* = 5) post final IASTM session. (**B**): Cage controls (*n* = 3) were maintained without intervention.

**Figure 2:**
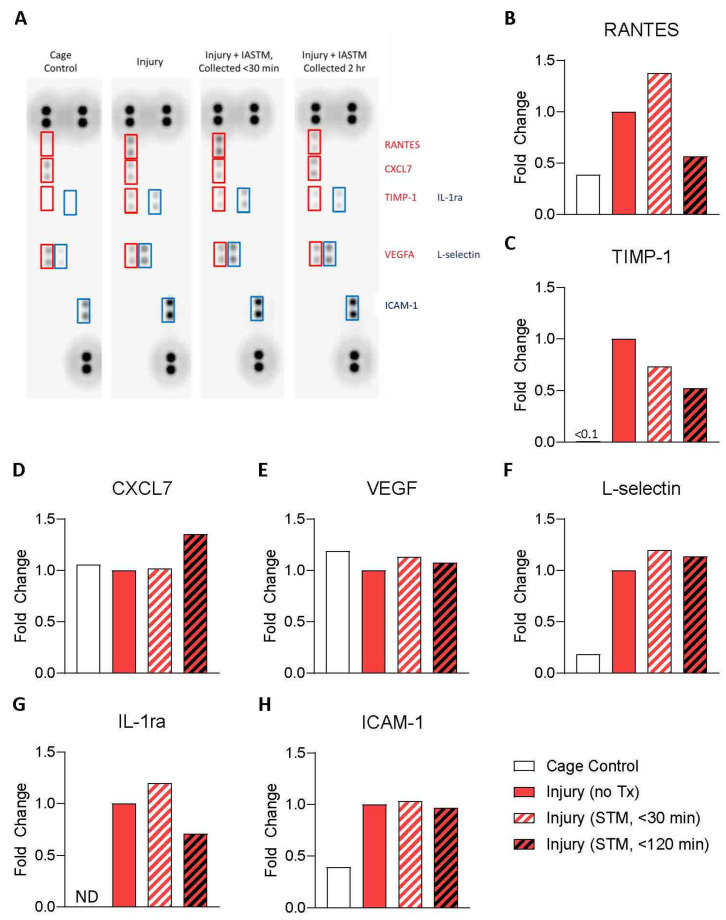
Membrane-based arrays for levels of select targets in muscle tissue homogenates. Homogenates were generated from muscle biopsies collected from cage controls or rats subjected to injury without treatment (untreated), injury plus IASTM with biopsy collected within 30 min of final IASTM, or injury plus IASTM with biopsy collected 2 h post-IASTM. Samples were pooled within treatment conditions with representative images of array results in (**A**). The data are expressed as fold change relative to untreated injury control for RANTES (**B**), TIMP-1 (**C**), CXCL-7 (**D**), VEGF (**E**), L-selectin (**F**), IL-1ra (**G**), and ICAM-1 (**H**). For TIMP-1, the value for the cage control group is presented in text. For IL-1ra, the signal was not detected (ND) for the cage control group.

A general schematic of the animal model is presented in Figure 1. To examine the tissue-specific changes associated with IASTM, homogenates from muscle biopsies were pooled within treatment groups and subjected to membrane-based cytokine arrays, which examined the expression levels of nearly thirty targets simultaneously (Figure 2A). These assays detected seven targets in at least one condition, whereas the other targets were below the detection limit (Figure 2A). Of these seven, only RANTES/CCL5 (Figure 2B) and Tissue Inhibitor of Metalloproteinase (TIMP)-1 (Figure 2C) were altered by ≥50% in IASTM-treated samples compared to untreated injury controls, with a reduction in the levels of both cytokines in samples obtained 120 min following IASTM; the other targets—C-X-C Motif Chemokine Ligand (CXCL)-7 (Figure 2D), Vascular Endothelial Growth Factor (VEGF) (Figure 2E), L-selectin (Figure 2F), Interleukin (IL)-1 Receptor Antagonist (IL1-ra) (Figure 2G), and Intercellular Adhesion Molecule (ICAM)-1(Figure 2H)—did not meet this threshold for further analysis.

**Figure 3:**
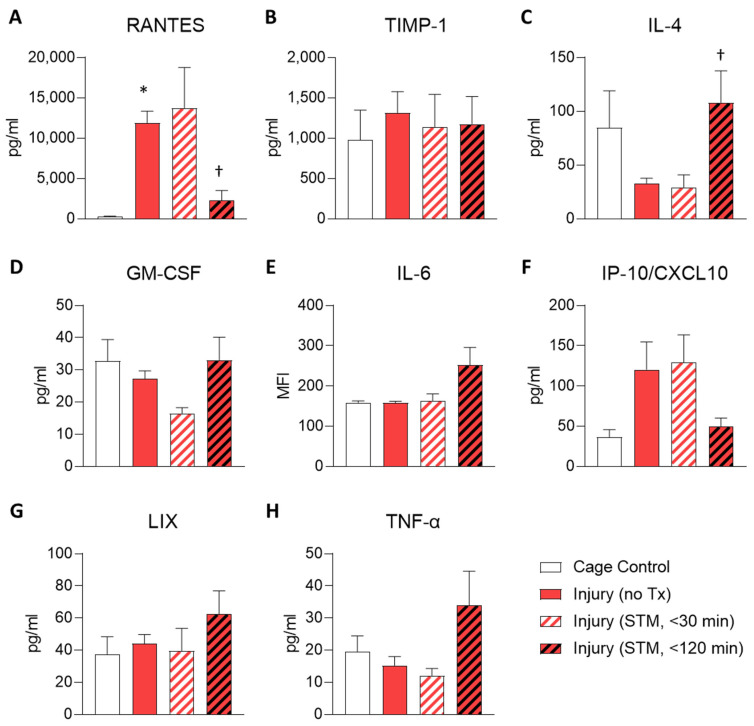
ELISAs for levels of select targets in muscle tissue homogenates. Homogenates were generated from muscle biopsies collected from cage controls or rats subjected to injury without treatment (untreated), injury plus IASTM with biopsy collected within 30 min of final IASTM, or injury plus IASTM with biopsy collected 2 h post-IASTM. Multiplex ELISAs were performed to quantify the levels of RANTES (**A**), TIMP-1 (**B**), IL-4 (**C**), GM-CSF (**D**), IL-6 (**E**), IP-10/CXCL10 (**F**), LIX (**G**), and TNF-α (**H**). *n* = 3 for cage controls and *n* = 5 for other treatment groups. The data are pg/mL (except IL-6 which is mean fluorescence intensity (MIF)) and expressed as mean ± SEM. Statistical testing was performed using a one-way ANOVA with Dunnett’s multiple comparison testing where * indicates *p* < 0.05 against cage control and † indicates *p* < 0.05 against untreated injury control.

Given that the results for the membrane arrays were generated using pooled samples, we next sought to validate the findings for RANTES/CCL5 and TIMP-1 using ELISA on individual samples to perform statistical testing. These results confirmed that the level of RANTES was higher in untreated injury samples, compared to cage controls, and was reduced within 120 min following IASTM (Figure 3A). In contrast, TIMP-1 levels were more variable between samples and did not reach statistical significance in this assay (Figure 3B).

ELISAs were also utilized to interrogate the levels of several other cytokines/chemokines of interest that were not detected on the membrane array, including IL-4, Granulocyte-Macrophage Colony Stimulating Factor (GM-CSF), IL-6, Intergeron-Inducible Protein (IP)-10/CXCL-10, CXCL-5/Lipopolysaccharide-Induced CXC Chemokine (LIX), and Tumor Necrosis Factor (TNF)-α (Figure 3C–H). Among these, only IL-4 levels were altered in samples subjected to IASTM compared to untreated injury controls, with levels of this cytokine increasing by approximately 3-fold in samples obtained 120 min following IASTM (Figure 3C).

## 3. Discussion

LBP is one of the most common problems affecting people of all ages and can be caused by a variety of factors, including injury, poor posture, and underlying medical conditions. This makes it difficult to determine the effective course of treatment, consequently making LBP challenging to manage. The prevalence and challenge of managing LBP has encouraged research into non-pharmacological and non-invasive therapies in an effort to find a less expensive, non-addictive, and yet effective form of treatment. The financial burden of healthcare costs associated with prescription medication and more invasive treatments, like surgery, may be reduced by alternatives like STM and massage. Although these alternatives are not one-size-fits-all treatments for LBP, these non-invasive treatments are known to have benefits for LBP such as reducing pain, improving range of motion, increasing circulation, etc. [4]. One of the potential mechanisms of these benefits is the regulation of inflammation, as suggested in our previous report [5]. Therefore, we further hypothesized that a better understanding of molecular changes of more inflammatory cytokines could open up wide adoption of STM and/or massage to manage LBP.

Our study analyzed tissue homogenates from muscle biopsies in several groups: uninjured cage controls, injured without treatment, injured + IASTM (sampled and collected within 30 min of treatment), and injured + IASTM (sampled and collected within 120 min of treatment). Our results indicated that this model of chronic LBP leads to elevated soft tissue levels of the chemokine RANTES and that IASTM decreased the levels of RANTES in rats within 120 min of treatment. This is consistent with a prior report demonstrating that IASTM decreased serum levels of RANTES in this same model, suggesting that injured tissue may be a major source of serum RANTES in this model [5]. Given that RANTES exerts generally pro-inflammatory effects [6], these findings provide important molecular-level information on a potential role for IASTM in reducing inflammation in vivo.

Additionally, we found that, at the same time point, IASTM increases tissue levels of IL-4, which has been shown to suppress the production of pro-inflammatory cytokines TNF-alpha and IL-1β while also stimulating the production of the IL-1 receptor antagonist (IL-1ra) [7]. IL-4 has also been linked to macrophage activation which counteracts inflammation by releasing IL-1ra, IL-10, and TGF-β [7]. Thus, our observation of increased tissue levels of IL-4 following IASTM supports a potential anti-inflammatory effect of this treatment modality. 

Notably, neither the present study nor the prior report detected changes in IL-6 or IL-10 levels in either the tissue or serum following IASTM [5]. Additionally, although our prior report demonstrated increased serum levels of NPY following IASTM, those results were obtained from animals subjected to a short, 3-day total IASTM treatment protocol rather than the 14-day protocol used here [5]. We did not allocate the limited resources in the present study to examine NPY since its levels at the 14-day time point did not differ between groups.

TIMP-1 is an inflammation-related protein that inhibits the activity of matrix metalloproteinases (MMPs), potentially preventing MMPs from breaking down the extracellular matrix [8]. TIMP-1 also has cytokine-line functions, such as binding to receptors on the surface of immune cells and regulating signaling pathways that influence cell behavior [8]. Many inflammatory diseases, including rheumatoid arthritis, asthma, and psoriasis, show upregulated levels of TIMP-1, suggesting that this protein could be a promising therapeutic target for the development of new anti-inflammatory treatments [9]. Our findings regarding TIMP-1 were variable between methodologies, but it is worth noting that this protein has mRNA splice variants which may alter the amino acid sequence [10,11]. Since the assays contained proprietary information, the manufacturers were unwilling to disclose details regarding the specific immunogen used to develop the antibodies; thus, we are unable to comment on the discrepancy between techniques. 

We additionally investigated tissue levels of several other proteins that have been associated with pro- or anti-inflammatory effects and may play a role in various types of LBP, including CXCL5/LIX (the murine homolog of IL-8), GM-CSF, ICAM-1, IL1-ra, IL-6, IP-10/CXCL10, TNF-α, and VEGF [12,13,14]. However, our analyses were unable to detect any IASTM-mediated effects on the levels of these cytokines in this model. These findings may be representative of the limitations of our current study (see below).

It is important to note that our study has several important limitations which may impact its generalizability. First, our design includes evaluation of only male rats at a single time point (two weeks post-injury) in a single model of chronic, induced LBP using Freund’s adjuvant. Thus, it is possible that these and/or other factors were altered earlier or later in the time course of induced LBP or with IASTM. Additionally, our analyses were performed on a relatively small sample size due to the availability of samples from a prior study. This potentially impacted our power to detect minor changes in specific factors between groups. And, although sham and IASTM-treated groups were handled and restrained identically, cage controls were not subjected to such intervention; therefore, it is formally possible that the observed increase in RANTES in untreated injured controls, compared to cage controls, was due to stress or some factor other than the LBP model. Finally, it is also possible that the effects observed in the tissue levels of RANTES and IL-4 were not the result of mechanical force per se, but were related to other aspects of the intervention, such as transient hyperemia or, since the rats were conscious, the mind–body aspects of massage. That said, taken with the observations of Loghmani et al. related to gait and circulating factors [5], our study is consistent with the notion that IASTM may exert anti-inflammatory effects in LBP and could be a promising treatment option for individuals suffering from musculoskeletal inflammation or injury. Future work is required to confirm this possibility.

## 4. Materials and Methods

### 4.1. Animal Model and Soft Tissue Biopsies

All soft tissue biopsies utilized in this study were collected from rats involved in a prior report and full details of the animal husbandry, injury model, and IASTM interventions may be found therein [5]. Briefly, adult male Sprague-Dawley rats (11 to 16 weeks old) were kept as cage controls (*n* = 3) or subjected to induced chronic inflammatory LBP through injection of 50 μL Complete Freund’s adjuvant (CFA) unilaterally while the animals were under isoflurane anesthesia. Samples discussed in the present study were obtained at sacrifice from cage controls or injured rats 14 days post-injection randomized to the following groups: (1) sham treatment (*n* = 5) or (2) three IASTM sessions/week to the region of injury over two weeks (6 IASTM sessions in total) (*n* = 10) was performed while conscious. All IASTM sessions were administered by a single examiner, who was trained and experienced in IASTM, using an IASTM device designed for treating small areas to manipulate the injured tissue [4]. Animals in both sham and treatment groups were handled the same—removal from the cage, covering of the head with a towel, and placed in a swaddling handhold as described previously. Sham treatment was carried out using a light stroke from a soft-bristled paint brush. The IASTM treatment (or sham) lasted 5 min/session at a pressure within the subject’s tolerance—for example, no withdrawal response, no fur pigmentation/discoloration, or vocalization—using an average force of 2.46 N *±* 0.42 N (i.e., 0.55 *±* 0.09 lbs) [5]. Rats were euthanized by asphyxiation using carbon dioxide, and immediately post-mortem, muscle biopsies were collected from the region of injury and subsequently snap frozen. Rats in Group 2 were sacrificed within 30 min post-IASTM (*n* = 5) or two hours post-IASTM (*n* = 5) to compare the immediate and delayed effects of soft tissue manipulation. All animal procedures were performed in alignment with a protocol approved by the Indiana University Institutional Animal Care & Use Committee and national standards.

Muscle biopsies were homogenized in 1X RIPA buffer (Cell Signaling, Danvers, MA, USA) with 1X Halt Protease and Phosphatase Inhibitor Cocktail (Thermo Fisher Scientific, Waltham, MA, USA) using a Bullet Blender (Next Advance, Troy, NY, USA). Protein concentration was determined using a BCA Assay (Thermo Fisher Scientific, Waltham, MA, USA) on a FilterMax F3 plate reader (Molecular Devices, San Jose, CA, USA).

### 4.2. Cytokine Membrane Array

Pooled tissue homogenates were analyzed using the Proteome Profiler Rat Cytokine Array Kit Panel A (R&D Systems, Minneapolis, MN, USA) as directed by the manufacturer and as reported previously [15]. This multiplex assay provided simultaneous measurement of the following targets: CCL3/MIP-1 alpha, CCL5/RANTES, CCL20/MIP-3 alpha, CNTF, CXC3CL1/Fractalkine, CXCL1/CINC-1, CXCL3/CINC-2 alpha/beta, CXCL2/CINC-3, CXCL7/Thymus Chemokine, CXCL9/MIG, CXCL10/IP-10, GM-CSF, ICAM-1, IFN-gamma, IL-1 alpha/IL-1F1, IL-1ra/IL-1F3, IL-2, IL-3, IL-4, IL-6, IL-10, IL-13, IL-17, L-Selectin, TIMP-1, TNF-alpha, and VEGF. Briefly, 80 µg total protein was pooled for each individual rat within the respective treatment group (*n* = 5 per treatment group except cage control where *n* = 3) resulting in a 400 µg total protein sample being loaded onto each membrane. The arrays were developed using WesternBright Quantum reagent (Advansta, San Jose, CA, USA) on a C-Digit scanner (LI-COR, Lincoln, NE, USA) and signal densities were determined using the Empiria Studio Version 2.3 software package (LI-COR). Data for each target were expressed relative to the average reference spot density on the respective membrane and normalized to the untreated sample.

### 4.3. Enzyme-Linked Immunosorbent Assays

Individual tissue homogenates were analyzed using a custom-made GeniePlex Multiplex Assay (AssayGenie, Dublin, Ireland) to quantify levels of CXCL10, GM-CSF, IL-4, IL-6, LIX, TNF-α, TIMP-1, and RANTES. The assay was performed as directed by the manufacturer, except 1X RIPA plus Halt Protease Inhibitor Cocktail was substituted for the tissue lysis buffer included with the kit. Assays were run on an Accuri C6 Flow Cytometer (Becton, Dickinson and Company, Franklin Lakes, NJ, USA) using 300 µg total protein per sample. Quantification of results was performed by AssayGenie using FCAP Array Software Version 3.0 (Becton, Dickinson and Company, Franklin Lakes, NJ, USA) by a scorer that was blinded to sample identity.

### 4.4. Statistical Analyses

Statistical analyses were performed using GraphPad Prism 9 (GraphPad Software, Boston, MA, USA) as described in each respective figure legend. A *p*-value of <0.05 was considered significant.

## 5. Conclusions

Our study found that IASTM is associated with reduced soft tissue levels of RANTES/CCL5 and increased soft tissue levels of IL-4 in rats with chronic, induced LBP. Combined with our prior report demonstrating that IASTM improved gait patterns in rats with induced LBP [5], the present findings suggest that IASTM exerts effects involving changes in pro-/anti-inflammatory cytokines in circulation and at the tissue level. Future studies are needed to confirm these findings in human subjects and to investigate the long-term effects of IASTM on soft tissue levels of pro- and anti-inflammatory cytokines.

## Data Availability

Datasets used and/or analyzed are available from the corresponding author on reasonable request.
